# Approaches to improve publication activity of surgical departments: Invited Commentary: “Publication activity of chief and senior general/visceral surgeons in German university hospitals - a ten-year analysis”, Böckmann et al.

**DOI:** 10.1007/s00423-021-02243-4

**Published:** 2021-06-24

**Authors:** Christoph W. Michalski

**Affiliations:** Department of General Surgery, University Hospital Ulm, Ulm, Germany

Scientific publication activity is one of the core tasks of academic surgeons and is the most measurable dimension of a department’s research endeavours. A transparent comparison of publication activity of General Surgery Departments at German university hospitals has been lacking so far. Böckmann and co-authors analysed academic output of these institutions between 2007 and 2017 in terms of overall number of publications and of cumulative impact factor (IF), as well as of the number of publications and the cumulative IF per member of the respective department. They demonstrate significant differences between departments, both in the cumulative IF metric and in the cumulative IF/staff surgeon.

Looking closer at these wide variations in publication activity, it appears that there are only few institutions with a high cumulative IF, but on the other hand a substantial number of institutions with a rather low cumulative IF. However, it must be considered that this analysis spans over a 10-year period, which may dilute some relevant changes over the last years in both ways, for the better and for the worse. Thus, it would be interesting to see a time trend analysis or the key figures of the departments over time.

Another interesting finding is the mean or median IF per publication, which was 3.0 or 2.7 respectively, demonstrating that the publication activity of German surgical departments is still largely present in the low to medium IF sector.

However, the important questions are what are the reasons for the generally relatively low publication activity? Has increasing economic pressure contributed to the moderate academic output? How has financial faculty contribution to General Surgery developed in the last decade? Is it associated with publication activity? And even more importantly: How can publication activity of German surgical departments be improved?

Learning from other disciplines, one chance for improvement would be to promote the possibilities for protected research time within surgical departments. A growing number of clinician scientist programs or faculty funding programs offer this opportunity. However, most of them are in large part exploited by other disciplines (e.g. internal medicine) for various reasons. One of the reasons is that only few residents apply for a spot in these programs because they fear to miss out on operating room time. Clinician scientist programs of German medical faculties also frequently aim at recruiting residents with a strong interest in “wet lab” research. Thus, there is a lack of structured programs for clinical research activities and the development of prospective trials. Such programs would relatively soon translate into higher quality clinical research with international impact — something that our neighbours in the Netherlands and in the Scandinavian countries have very successfully implemented.

